# Effects of Wheat Composition on the Physicochemical and Volatile Components of *Daqu* (A Primary Starter for Chinese *Baijiu* Fermentation)

**DOI:** 10.3390/foods14213638

**Published:** 2025-10-24

**Authors:** Huiyue Deng, Huiling Huang, Rong Yang, Kangjie Yu, Rui Liao, Yi Ma

**Affiliations:** 1 College of Bioengineering, Sichuan University of Science and Engineering, Yibin 644000, China; 2 Engineering Technology Research Center of Special Grain for Wine Making, Yibin 644000, China; 3 State Key Laboratory for Crop Stress Resistance and High-Efficiency Production, College of Agronomy, Northwest A&F University, Xianyang 712100, China

**Keywords:** wheat varieties, medium-high-temperature *Daqu*, physicochemical properties, quality characteristics, volatile components

## Abstract

Wheat serves as the primary raw material for medium- and high-temperature *Daqu*, a key saccharification, fermentation, and flavoring agent in Chinese *Baijiu* production. To investigate the influence of wheat’s main components on the quality and volatile profile of *Daqu*, this study comparatively analyzed the main components of three distinct wheat varieties and the physicochemical indices and volatile characteristics of the *Daqu* produced from them. Results indicated significant differences (*p* < 0.05) in the main components of the wheats and in the physicochemical indices and sensory scores of their corresponding *Daqu* samples. CM605 *Daqu* exhibited the highest acidity, NM660 *Daqu* showed the highest saccharification power, and MM907 *Daqu* had the highest liquefaction power. A total of 66 volatile compounds were identified across the three *Daqu* types, encompassing esters, alcohols, aldehydes, acids, phenols, ketones, pyrazines, etc. The contents of volatile compounds varied significantly among the *Daqu* samples made from different wheats; notably, the total contents of esters, alcohols, and aldehydes showed significant differences (*p* < 0.05). The Spearman correlation analysis revealed highly significant correlations (*p* < 0.01) between several wheat quality characteristics and *Daqu* quality indices, identifying wheat starch and protein as key factors affecting *Daqu* quality. The PLS-DA analysis revealed 34 differential volatile compounds. Mantel test correlation analysis further confirmed that wheat components including starch, albumin, globulin, gliadin, glutenin, and fat could influence the differential volatiles in *Daqu*. For instance, amylopectin content showed a significant positive correlation with n-pentanol, isovaleric acid, and propyl acetate (*p* < 0.05). This study provides a solid foundation for a deeper understanding of the relationship between wheat composition and *Daqu* quality, facilitating the more precise selection of wheat varieties to improve *Daqu* quality during production.

## 1. Introduction

Chinese *Baijiu*, one of the world’s six major distilled spirits, boasts over 2000 years of history and is deeply ingrained in Chinese culture. It is produced from grains using *Daqu*, a traditional brick-shaped microbial starter culture, as the key saccharification, fermentation, and flavor-producing agent. *Baijiu* relies on *Daqu* to provide functional microorganisms and complex enzymes that catalyze the biochemical reactions during fermentation [1]. In this process, starch is enzymatically degraded into reducing sugars, which subsequently serve as substrates for microbial biosynthesis of ethanol, lactic acid, and ester compounds. By the end of 2022, China’s *Baijiu* enterprises above the designated size recorded a total production output of 6.7124 million kiloliters, representing a year-on-year (y-o-y) decrease of 5.58%. The number of such enterprises stood at 963. They achieved cumulative product sales revenue of RMB 662.645 billion (up 9.64% y-o-y) and realized cumulative profits of RMB 220.172 billion (up 29.36% y-o-y). (Data sourced from the National Bureau of Statistics of China and the China Alcoholic Drinks Association). The annual export volume was 720 million US dollars (Data from the General Administration of Customs of China), mainly to South Korea, ASEAN, and European and American Chinese communities. As the core saccharification and fermentation agent in traditional Chinese liquor (*Baijiu*) brewing, *Daqu* quality critically influences the brewing efficiency and determines the flavor profile and overall quality of the final product [2,3]. Wheat, being the primary raw material for *Daqu* production, plays a critical role in determining the functional properties of *Daqu* during *Baijiu* fermentation [4,5]. Therefore, investigating the relationship between wheat, as the key raw material, and *Daqu* is essential to optimize *Daqu* production processes, enhance *Baijiu* quality, and promote the sustainable development of the traditional liquor industry.

The content and compositional ratio of key wheat components (starch, protein, fat, and so on) critically shape *Daqu* quality attributes, concurrently governing the formation mechanisms of flavor compounds in the final *Baijiu*. For instance, soft wheat—characterized by a higher starch content but lower protein content compared to hard wheats—directly influences the microbial metabolic landscape by altering the carbon-to-nitrogen (C/N) ratio. The abundant starch (a carbon source) primarily fuels microbial growth and energy metabolism (e.g., glycolysis), leading to the production of foundational metabolites like sugars and ethanol. Conversely, while a lower protein content might limit nitrogen availability, it is precisely the balance between starch (carbon) and protein (nitrogen) that is critical. Studies indicate that an optimal proportion of 15% soft wheat yields more complete *Daqu* fermentation, accompanied by significantly higher concentrations of volatile flavor compounds such as esters and pyrazines compared to other formulations [6]. Previous research demonstrated that *Daqu* produced with wheat milled at 55% fineness (i.e., 55% of the particles by weight did not pass through a 20-mesh sieve) significantly enhanced roasted and sauce-like aromas in high-temperature *Daqu* [7]. Comparatively, studies on *Daqu* production suggest that variegated-colored wheat (locally termed ‘Hua Mai’) proved superior suitability for medium-high temperature *Daqu* compared to white wheat (‘Bai Mai’). This distinction is likely attributed to its higher bran phenolic content [8]. Consequently, the quality attributes of raw materials profoundly influence *Daqu* properties. Current evidence confirms that variations in wheat composition and characteristics driven by cultivar differences and geographical origins which can directly modulate *Daqu*’s metabolic activity and flavor development [9]. Recent research on wheat as a fermentation substrate has primarily focused on: (1) comparisons of wheat physicochemical properties, processing characteristics, and raw material flavor profiles; (2) microbial community analysis of *Daqu*; and (3) quality assessment of *Daqu* itself. However, the influence of wheat quality and composition on *Daqu* fermentation requires deeper investigation. Currently, breweries predominantly utilize blended wheat varieties for *Daqu* production, with single-variety applications remaining limited. Research specifically addressing the effects of wheat quality and compositional variations on *Daqu* fermentation processes remains insufficient.

The three wheat varieties (Chuanmai 605, Mianmai 907, and Nanmai 660) were selected based on a preliminary cluster analysis of physicochemical properties (e.g., starch, protein, gluten content, hardness) among predominant Sichuan wheats. The analysis showed that these three varieties belonged to distinct clusters, exhibiting significant divergence and representativeness in their profiles. This pre-selection strategy was designed to maximize the raw material quality gradient, thereby effectively revealing variety-induced effects on *Daqu* quality.

This study aimed to analyze the impact of these wheat compositional variations on *Daqu* flavor compounds and to investigate the relationship between raw material constituents and microbial metabolism within *Daqu*. The research provides a theoretical basis for cultivating high-quality *Daqu*-specific wheat, selecting optimal varieties, and standardizing *Daqu* quality parameters.

Regarding their breeding background, Chuanmai 605 (CM605) was bred by the Crop Research Institute of the Sichuan Academy of Agricultural Sciences. Its pedigree, ICA80/99-6890//Chuanmai 44, reflects a composite crossing strategy that integrated introduced germplasm (ICA80 from CIMMYT) with local germplasm to combine high yield, stripe rust resistance, and adaptability to Sichuan’s conditions; it was approved in 2020. Similarly, Mianmai 907 (MM907) and Nanmai 660 (NM660) were developed by local Sichuan institutions (e.g., Mianyang Academy of Agricultural Sciences) with breeding objectives focused on high yield, stability, disease resistance, and adaptability for the Sichuan Basin.

In terms of their quality profiles, these three varieties represent divergent potentials for end-use applications. Chuanmai 605 (CM605), as a soft, weak-gluten wheat, is typically associated with the processing quality of foods like biscuits and pastries due to its low protein content and weak gluten strength. Nanmai 660 (NM660), as a hard, medium-gluten wheat, possesses higher protein and wet gluten content, aligning its quality traits with the requirements for high-quality steamed bread and noodles. Mianmai 907 (MM907), with its intermediate characteristics, offers broad adaptability. However, their potential application and suitability in the production of brewing *Daqu* have not been systematically investigated. Therefore, this study selected this combination of varieties with a significant quality gradient to provide crucial theoretical evidence and practical guidance for screening and breeding wheat specifically dedicated to *Daqu* production.

The research analyzed the impact of these compositional variations on *Daqu* flavor compounds and investigated the relationship between raw material constituents and microbial metabolism within *Daqu*. This research aims to provide a theoretical basis for cultivating high-quality wheat suitable for *Daqu* production, selecting optimal wheat varieties, and standardizing *Daqu* quality parameters.

## 2. Materials and Methods

### 2.1. Research Materials and Experimental Design

Three predominant commercial wheat varieties from Sichuan Province were investigated: Chuanmai 605 (CM605, soft, weak-gluten tendency), Mianmai 907 (MM907, intermediate hardness), and Nanmai 660 (NM660, hard, medium-gluten). CM605 was provided by Chengdu Damei Seed Industry Co., Ltd. (Chengdu, Sichuan, China), while MM907 and NM660 were sourced from Sichuan Taiwo Seed Industry Co., Ltd. (Mianyang, Sichuan, China). To ensure comparability, all wheat samples were from the same harvest year (2024), thereby controlling for potential inter-annual environmental variations. 10 kg of each variety of wheat.

The experiment was arranged in a completely randomized design. Each wheat variety constituted an independent treatment. To ensure statistical reliability, three biological replicates were performed per treatment, meaning the entire *Daqu*-making process was independently repeated three times for each variety.

### 2.2. Daqu Production Process

**Figure 1:**
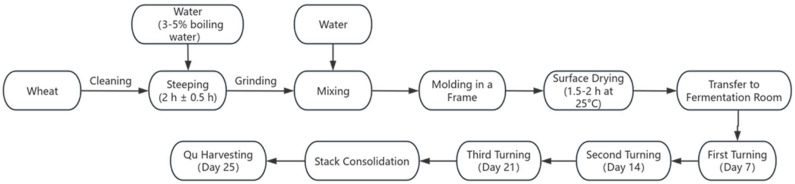
Production process of *Daqu*.

Medium-high temperature *Daqu* was produced following traditional manual methods at Yibin Nanxi Wine Industry Co. (Yibin, Sichuan, China) The process was as follows: wheat grains were conditioned by spraying with 3~5% boiling water (used for surface sterilization and improved water absorption), thoroughly mixed, and then piled for 2 h ± 0.5 h to allow uniform water penetration. The steeped wheat was crushed using a small roller crusher to achieve the desired state of a fragmented endosperm while preserving the pericarp integrity. The crushed material was mixed with 38% ± 2% water. The mixture was then molded into bricks (300 × 190 × 80 mm^3^) within a frame by compaction. The raw Qu bricks were air-dried at 25 °C until the surface became non-sticky (surface drying for approximately 1.5–2 h). All batches subsequently underwent a 25-day fermentation period in the same room with three turnings on days 7, 14, and 21 (Figure 1).

### 2.3. Wheat Quality Characteristics and Major Component Analysis

The quality characteristics and major components of wheat were determined following the corresponding Chinese National Standards (GB/T). Wheat test weight was measured using HGT-1000 according to GB/T 5498-2013 Inspection of Grain and Oils—Determination of Test Weight [10]. Wheat hardness was determined via hardness index tester per GB/T 21304-2007 Determination of wheat hardness—Hardness index method [11]. Thousand-kernel weight (dry basis) was analyzed following GB/T 5519-2018 Cereals and Pulses—Determination of the Mass of 1000 Grains [12]. Wet gluten content was quantified in accordance with GB/T 5506.2-2008 Wheat and Wheat Flour—Gluten Content—Part 2: Instrumental Determination of Wet Gluten [13]. Moisture content was determined by direct drying method as per GB 5009.3-2016 National Food Safety Standard—Determination of Moisture [14], pulverized samples (5.000 ± 0.0001 g) were dried at 101–105 °C to constant weight. Weight loss percentage was calculated. Crude fat content was measured via Soxhlet extraction based on GB 5009.6-2016 National Food Safety Standard—Determination of Fat [15]. Crude protein content was analyzed according to GB 5009.5-2016 National Food Safety Standard—Determination of Protein using the Kjeldahl method [16]. Wheat starch content was determined according to the acid hydrolysis method specified in Chinese National Standard GB 5009.9-2023 National Food Safety Standard—Determination of Starch in Foods [17]. Amylose and amylopectin contents in wheat were quantified following Chinese National Standard GB 7648-1987 Method for Determination of Straight-Chain Amylose in Rice, Maize and Cereal Grains [18]. Wheat albumin, globulin, gliadin, and glutenin content were determined using the Osborne fractionation method based on Wang et al. [19].

### 2.4. Physicochemical Property Analysis of Daqu and Sensory Evaluation of Daqu

Moisture, titratable acidity, amino acid nitrogen, saccharification power, and liquefaction power of *Daqu* were determined according to QB/T 4257-2011 Provincial Standard of Liaoning: General Methods of analysis for *Daqu* [20]. Moisture was determined by oven-drying at 105 ± 2 °C to constant weight. Acidity was measured by titrating aqueous extracts with 0.1 mol/L NaOH. Amino acid nitrogen was quantified via formol titration. Saccharification power was assessed by measuring reducing sugars (as maltose) released from starch using the Fehling method. Liquefaction power was evaluated by timing the disappearance of the blue color in the starch-iodine complex. Sensory evaluation was conducted according to standardized procedures by trained panelists.

A sensory evaluation panel consisting of five experienced *Daqu* production technicians from the brewery’s technical center was employed. The panelists were proficient in sensory analysis and *Daqu* quality assessment. For the evaluation, we prepared samples from three independent *Daqu* production batches (biological replicates) for each of the three wheat varieties (CM605, NM660, MM907), resulting in a total of 3 varieties × 5 replicates = 15 *Daqu* bricks. From each brick, 200 g of sample, including both crust and core sections, were placed on labeled A4 sheets. Each sample was coded with a random three-digit number. The 15 samples were presented to each evaluator in a randomized order across multiple sessions, with two samples per session and a 10 min interval between assessments. Evaluators received two randomly presented samples per session with 10 min intervals between assessments. Following the China Light Industry Standard QB/T 4259-2011 Strong-Flavor *Daqu* [21], evaluators first scored appearance, aroma, cross-section characteristics, and shell thickness, then provided a comprehensive score for each sample. Specific scoring criteria are detailed in Table 1.

### 2.5. Determination of Volatile Compounds in Daqu

Volatile compounds in *Daqu* were determined using Headspace Solid-Phase Microextraction coupled with Gas Chromatography-Mass Spectrometry (HS-SPME-GC-MS), referencing the method of Li et al. [22]. with critical modifications. Briefly, 4 g of *Daqu* sample was ultrasonically extracted (130 W, 30 min) in 10 mL of saline solution after incubation at 4 °C overnight. The extract was then centrifuged (10,000 rpm, 20 min) and filtered. For HS-SPME, 5 mL of the supernatant was transferred to a 15 mL vial containing 1.5 g of NaCl. The vial was spiked with 30 μL of amyl acetate (boiling point 142 °C; 87.162 mg/mL) as an internal standard, which was selected for its improved coverage of high-boiling-point esters. After pre-equilibration at 50 °C, volatile compounds were extracted using a 50/30 μm DVB/CAR/PDMS fiber for 45 min. The fiber was then injected into the GC inlet at 250 °C for thermal desorption. Separation was achieved on a DB-WAX column (60 m × 0.25 mm × 0.25 μm) with a helium carrier gas flow rate of 1.2 mL/min. The oven temperature program was as follows: held at 45 °C for 3 min, ramped to 150 °C at 4 °C/min and held for 2 min, then ramped to 200 °C at 6 °C/min, and finally ramped to 230 °C at 9 °C/min and held for 10 min. Mass spectrometry detection was performed in electron impact (EI) mode at 70 eV, with the ion source and quadrupole temperatures set at 230 °C and 150 °C, respectively. The mass scan range was set to m/z 50–350 to enhance sensitivity for target volatiles. Compound identification required meeting the following three criteria: (1) mass spectral similarity >80% against the NIST 17a and Wiley libraries; (2) a calculated retention index (RI) deviation of less than 5% compared to literature values using a C7-C30 alkane series; and (3) the relative abundance of characteristic ions matching within a 15% tolerance compared to the reference standard or library spectrum.

### 2.6. Statistical Analysis

All measurements were performed in triplicate. Data were expressed as mean ± standard deviation (SD). Significant differences among groups were assessed by one-way analysis of variance (ANOVA) followed by the Least Significant Difference (LSD) post hoc test using SPSS software (version 26.0). Different letters above the bars in figures or in table superscripts (a, b, c) denote statistically significant differences (*p* < 0.05).

A partial least squares-discriminant analysis (PLS-DA) was performed to visualize the overall quality differences among *Daqu* samples from different wheat varieties and to identify key discriminant biomarkers.

For correlation analyses, the choice of statistical tests was based on the data structure. Spearman’s rank correlation analysis, performed with OriginPro2025, was applied to assess the monotonic relationships between individual wheat components and individual *Daqu* physicochemical parameters. This non-parametric method was chosen for its robustness without assuming normal data distribution. Conversely, the Mantel test was employed to examine the overall correlation between the multivariate distance matrices of wheat nutrient composition and *Daqu* volatile compounds, testing the hypothesis that wheat samples with similar nutrient profiles yield *Daqu* with similar volatile fingerprints.

## 3. Results and Discussion

### 3.1. Impact of Wheat Composition on Physicochemical Properties of Daqu

The dry basis thousand kernel weight, bulk density, hardness index, and wet gluten content of the three brewing wheat varieties were determined, with results shown in Table 2. Significant differences existed in the characteristics among wheat varieties:

NM660 exhibited significantly higher dry basis thousand kernel weight (50.59 g) and wet gluten content (26.34%) than the other samples, and a significantly higher hardness index (48.43%) than CM605 and MM907 (*p* < 0.05). CM605 had significantly higher bulk density (825.67 g/L) than MM907, but significantly lower dry basis thousand kernel weight (44.44 g), hardness index (44.24%), and wet gluten content (24.13%) than NM660 (*p* < 0.05). MM907 showed significantly lower bulk density (786.00 g/L) than CM605 and NM660 (*p* < 0.05). NM660 belonged to the medium-gluten wheat category, while CM605 and MM907 fell into the low-gluten category; CM605 was classified as soft wheat, with MM907 and NM660 being intermediate between soft and hard wheat.

The major components and contents of different wheat varieties are shown in Table 3. CM605 had significantly higher starch content (67.97%) and amylose content (26.04%) but significantly lower amylopectin content (73.96%), albumin content (18.68 mg/g), globulin content (14.82 mg/g), and total protein content (113.30 mg/g) than the other wheat samples (*p* < 0.05). Its moisture content (12.10%) was significantly lower than that of all other samples. MM907 had significantly higher amylopectin content (82.69%), fat content (1.89%), and moisture content (13.91%) than the other wheat samples (*p* < 0.05). NM660 had significantly higher albumin content (27.84 mg/g), glutenin content (54.74 mg/g), and total protein content (140.15 mg/g) than the other samples, but significantly lower fat content (0.64%) than CM605 and MM907 (*p* < 0.05).

The physicochemical indexes of *Daqu* can reflect the quality of *Daqu* to a certain extent (Table 4).

To inhibit contaminating bacteria and prevent mold, according to QB/T 4259-2011 “Intense Fragrance *Daqu*”, the moisture content of finished *Daqu* should be less than 14%. The moisture content of CM605 and NM660 *Daqu* samples complied, and a significant difference existed between them (*p* < 0.05). The acidity of *Daqu* is mainly produced by microorganisms through the tricarboxylic acid (TCA) cycle via organic acid metabolism by degrading starch, fat, and protein [23]. Excessively high acidity masks the flavor, while excessively low acidity is not conducive to flavor presentation [24]. The acidity of the three types of *Daqu* differed significantly but was within the appropriate range, with CM605 *Daqu* being significantly higher (*p* < 0.05). Amino acid nitrogen is an important index of the aroma-producing potential [25]. The amino acid nitrogen content of NM660 *Daqu* was significantly higher (*p* < 0.05), indicating a high degree of utilization of proteins and peptides. The saccharification power and liquefaction power directly reflect the ability of enzymes produced by microorganisms to convert starch into fermentable sugar [26]. QB/T 4259-2011 stipulates saccharification power between 100~1000 mg/g·h and liquefaction power ≥ 0.20 g/g·h. The values for the three *Daqu* were within ranges. NM660 sample had the highest saccharification power, and MM907 sample had the highest liquefaction power, with significant differences (*p* < 0.05), indirectly reflecting the abundance and metabolism of microorganisms.

**Figure 2:**
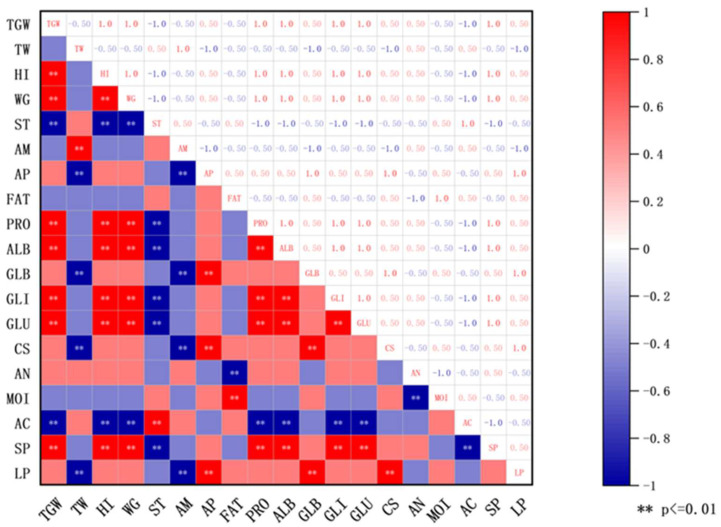
Physicochemical correlation between quality of *Daqu* and wheat. ** *p* < 0.01. Abbreviations: TGW: Wheat thousand grain weight; TW: Wheat test weight; HI: Wheat hardness index; WG: Wheat wet gluten content; ST: Total starch content; AM: Amylose content; AP: Amylopectin content; FAT: Fat content; PRO: Wheat total protein content; ALB: Albumin content; GLB: Globulin content; GLI: Gliadin content; GLU: Glutenin content; CS: *Daqu* comprehensive score; AN: *Daqu* amino acid nitrogen; MOI: *Daqu* moisture content; AC: *Daqu* acidity; SP: *Daqu* saccharification power; LP: *Daqu* liquefaction power.

Spearman correlation analysis (Figure 2) further revealed significant statistical relationships between principal wheat components and *Daqu* quality indicators: the comprehensive score of *Daqu* showed a highly significant positive correlation with wheat amylopectin content (*p* < 0.01) and a highly significant negative correlation with wheat amylose content (*p* < 0.01); the saccharification power of *Daqu* was highly significantly positively correlated with the contents of wheat total protein, albumin, gliadin, and glutenin (*p* < 0.01); the liquefaction power of *Daqu* showed a highly significant positive correlation with wheat amylopectin content and globulin content (*p* < 0.01), and a highly significant negative correlation with amylose content (*p* < 0.01). Furthermore, wheat bulk density showed a significant negative correlation with liquefaction power (*p* < 0.01), potentially as the porous structure of low bulk density wheat improves oxygen permeability [27]; the acidity of *Daqu* was highly significantly positively correlated with wheat total starch content (*p* < 0.01), while highly significantly negatively correlated with the contents of wheat total protein, albumin, gliadin, and glutenin (*p* < 0.01); wheat fat content demonstrated a highly significant positive correlation with *Daqu* moisture content (*p* < 0.01), likely due to the hydrophobicity of fat aiding moisture retention [28].

The physicochemical properties of *Daqu* essentially represent the expression of raw material components realized through microbial metabolism. CM605 *Daqu* showed the significantly highest acidity, highly related to its high amylose content (67.97%)—aligning with prior research abundant starch provided sufficient carbon source for microorganisms to metabolize organic acids (e.g., hexanoic acid, isovaleric acid, acetic acid) via the TCA cycle and other pathways [29]. NM660 *Daqu* had the significantly highest amino acid nitrogen, attributed to its high protein content (140.15 mg/g, highest total protein, albumin, and glutenin). Rich proteins were degraded by microorganisms during production, generating abundant free amino acids and small peptides. This explains the potential for higher alcohol production (via the Ehrlich pathway) and aldehyde formation (via Strecker degradation) [30,31], reflecting its “strong flavor-producing capacity.” The highest saccharification power of NM660 *Daqu* may be indirectly related to its high protein content, as the abundant nitrogen source likely supported a more robust microbial community capable of secreting hydrolytic enzymes [32]. MM907 *Daqu* showed the highest liquefaction power, which could be linked to its high amylopectin content (82.69%). The structure of amylopectin may present a more accessible substrate for amylolytic enzymes, potentially favoring their activity [33].

### 3.2. Impact of Wheat Composition on Sensory Properties of Daqu

**Figure 3:**
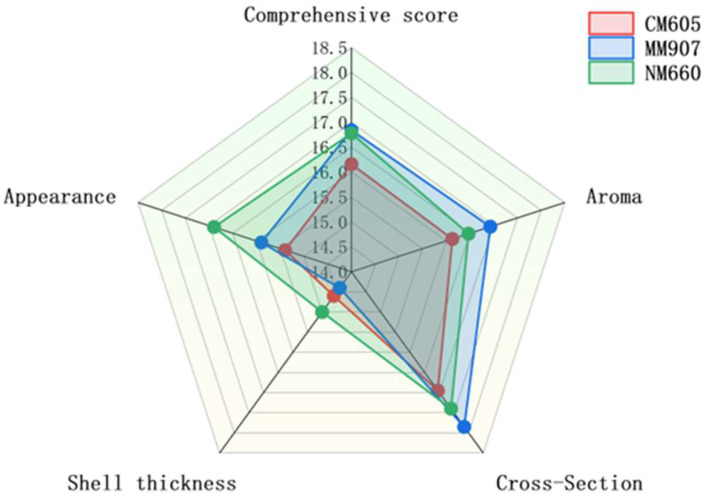
Sensory scores of *Daqu* from different wheat varieties.

The sensory evaluation profiles of *Daqu* produced from the three wheat varieties are presented in Figure 3. All *Daqu* types met the standard for second-grade classification. The radar chart illustrates distinct sensory profiles associated with each variety. NM660-*Daqu* was characterized by its superior performance in appearance and crust thickness, a critical quality attribute as the *Daqu* surface serves as the primary site for diverse microbial colonization and enzymatic activity. MM907-*Daqu* achieved the highest comprehensive score, leading in aroma and also performing well in cross-section evaluation. CM605-*Daqu*, while meeting the grade standard, received a comparatively lower comprehensive score. These observed sensory differences can be attributed to the physicochemical properties of the raw wheat materials. The higher hardness and gluten content of NM660 wheat likely contributed to the formation of a physical structure more conducive to microbial growth during *Daqu*-making [34], consistent with its superior scores in appearance and crust thickness. For a detailed statistical analysis of significant differences between varieties for each attribute, please refer to Supplementary Table S1.

### 3.3. Impact of Wheat Composition on Volatile Compounds in Daqu

A total of 66 volatile substances were detected using HS-SPME-GC-MS (Table 5), including esters (16), alcohols (15), aldehydes (8), acids (6), phenols (6), ketones (4), pyrazines (8), and others (3). Significant differences existed in the relative abundance and content.

**Figure 4:**
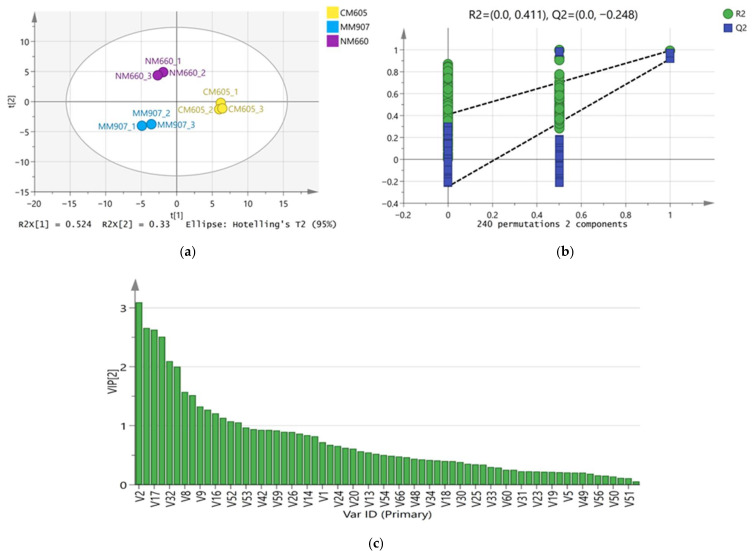
PLS-DA analysis of the volatile substances in *Daqu*. (**a**) Plot of PLS-DA scores; (**b**) Plot of substitution tests; (**c**) Plot of VIP scores.

To resolve the overall differences in volatile compounds among different *Daqu* and screen for key differential markers, Partial Least Squares-Discriminant Analysis (PLS-DA) was employed. The model parameters were satisfactory (R2X(cum) = 0.823, R2Y(cum) = 0.985, Q2(cum) = 0.958), and permutation test results (R2 = 0.411, Q2 = −0.248) indicated the model was not overfitted and possessed accurate discrimination and predictive ability. As shown in the score plot (Figure 4a), the three *Daqu* samples showed distinct clustering within the 95% confidence interval, indicating significant differences in their overall volatile compound profiles: NM660 and MM907 *Daqu* were separated from CM605 *Daqu* along the first principal component (PC1), while NM660 *Daqu* was separated from MM907 and CM605 *Daqu* along the second principal component (PC2). Using Variable Importance in Projection (VIP > 1), 34 key differential volatile compounds were further screened (Figure 4c). These compounds, quantified by weight, including 6 esters, 7 alcohols, 5 aldehydes, 4 acids, 2 phenols, 2 ketones, 5 pyrazines, and 3 others, which were the main contributors to the inter-group differences.

**Figure 5:**
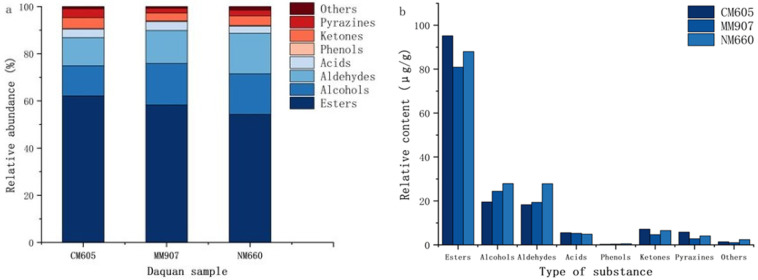
Volatile sµbstance content of *Daqu* proportion of different volatile compounds (**a**); relative content of various volatile compounds (**b**).

Esters affect flavor and serve as key quality indicators [35], accounting for 54.28% to 62.14% of total volatiles (Figure 5a). The total ester content was significantly higher in CM605 *Daqu* (Figure 5b), and Table 5 indicates significant variability (*p* < 0.05) in ester composition. Dominant esters included ethyl hexanoate (25.87 µg/g), ethyl butyrate (17.53 µg/g), and ethyl propionate (15.78 µg/g). Short-chain esters such as ethyl hexanoate impart fruity notes [6]. Higher fatty acid esters, which contribute to aroma retention, ranged from ethyl oleate (0.25–0.35 µg/g) and ethyl linoleate (0.43–0.54 µg/g) to ethyl palmitate (0.61–1.54 µg/g); these compounds impart no distinct aroma but influence persistence [36]. Several esters were unique to specific *Daqu* samples: ethyl laurate was detected exclusively in NM660, whereas isoamyl acetate and propyl acetate were found only in CM605.

Alcohols constitute primary flavor components [37], accounting for 12.76% to 17.60% (Figure 5a). Total alcohol content in NM660 and MM907 *Daqu* was significantly higher than in CM605 (Figure 5b). Table 5 shows significant differences (*p* < 0.05) in relative contents. Produced mainly by yeast metabolism of sugars and amino acids [38], key examples include n-pentanol (fruity), 1-octen-3-ol (imparting mushroom, floral, and hay-like notes), and phenethyl alcohol (fruity and slightly pungent) [39]. Higher alcohols (e.g., benzyl alcohol, isobutanol) harmonize aroma, enhance sweetness, prolong aftertaste [40]. Regarding unique compounds, furfuryl alcohol (caramel-like) was exclusive to NM660, and 1-hexanol (grassy) [41] was unique to MM907.

Aldehydes serve as important aroma components and acetal precursors [42]. Moderate levels enhance complexity and layering [43]. They accounted for 11.95% to 13.96% (Figure 5a). NM660 *Daqu* had significantly higher total aldehyde content (Figure 5b). Table 5 shows significant differences (*p* < 0.05). Benzaldehyde was most abundant. Hexanal content varied significantly, being highest in NM660 (9.43 µg/g) and lowest in CM605 (2.88 µg/g). Benzaldehyde provides almond-like notes [44], hexanal imparts grassy nuances [45]. Decanal and 2-pyrrole formaldehyde were detected exclusively in CM605.

Ketones were related to microbial fatty acid β-oxidation and coordinated flavor. They accounted for 3.36% to 4.66% (Figure 5a), with CM605 having the highest total ketone content (Figure 5b). Table 5 shows significant differences (*p* < 0.05). The highest content was found in 2-octanone (fruity), and then 2-heptanone. 1-Octen-3-one (grassy aroma [46] was found only in CM605.

Acids are important flavor components and precursors [47]. They accounted for 3.02% to 3.79% (Figure 5a). However, there was no significant difference in total acid content (Figure 5b). Table 5 shows significant differences (*p* < 0.05) in relative contents. Hexanoic acid, isovaleric acid, acetic acid higher. Propionic acid was detected only in CM605.

Phenolics produced by microbial metabolism [48]. Low proportion in total volatiles (Figure 5a). There was no significant difference in total phenolic content (Figure 5b). Table 5 shows 4-ethylguaiacol and 4-vinylguaiacol contributed smoky and floral notes. 4-Ethylguaiacol and 2,4-di-tert-butylphenol higher. Vanillin was detected only in CM605.

Pyrazines contribute to nutty, roasted, and baked aromas, but it is important to note that these aroma characteristics can also originate from other volatile compounds. Conversely, pyrazines themselves also contribute to a wider spectrum of aromas beyond these notes [49,50]. They accounted for 2.01% to 3.79% (Figure 5a). No significant difference in total pyrazine content (Figure 5b). Table 5 shows significant differences (*p* < 0.05). Other volatiles were limited.

Significant differences in the composition of volatile compound families were closely associated with the composition of the raw wheat materials, suggesting distinct underlying microbial metabolic processes.

Esters (54.28–62.14%) are primarily known to be produced by microbial esterification. The high starch content of CM605 (67.97%) could have promoted yeast glycolysis, potentially leading to acetyl-CoA accumulation that may have facilitated ester synthesis [51]. This is consistent with the significantly higher ester percentage in CM605 *Daqu* (62.14%) compared to that in MM907 (54.28%) and NM660 (57.15%) *Daqu*. The significantly lowest dry basis thousand-kernel weight (44.44 g), hardness index (44.24%), and wet gluten content (24.13%) of CM605 wheat may imply that differences in grain structure influenced the microbial environment during *Daqu*-making, potentially favoring ester-producing microorganisms.

Alcohols (12.76–17.60%) are mainly generated via glycolysis and amino acid metabolism in microbial systems. NM660 wheat had the highest total protein content. This abundant protein supply could have provided sufficient precursors for yeast to produce alcohols via the Ehrlich pathway [52]. The high moisture and high branched-chain starch content of MM907 may have created a more favorable environment for yeast growth and sugar metabolism.

Aldehydes (11.95–13.96%) are primarily derived from the Strecker degradation of amino acids and lipid oxidation. The highest protein content in NM660 wheat implies a rich pool of amino acid precursors, which can be converted to aldehydes through Strecker degradation [53].

Ketones (3.36–4.66%) mainly originate from the β-oxidation of fatty acids. The high starch content in CM605 wheat likely provided sufficient energy for microorganisms to facilitate the β-oxidation pathway, which might explain the increased ketone production.

Although no significant differences were observed in the percentages of acids, phenolics, and pyrazines, the exclusive detection of propionic acid and vanillin in CM605 *Daqu* implied distinct metabolic pathways in this variety.

Overall, the data suggest that differences in the chemical composition of the wheat raw materials regulated microbial metabolism, altered the profile of volatile components, and ultimately resulted in the observed variations. The PLS-DA model confirmed the holistic nature of this variation, while the Mantel Test analysis statistically quantified the correlations between specific wheat components and key flavor compounds, further supporting the proposed link between raw material composition and *Daqu* volatile profiles.

**Figure 6:**
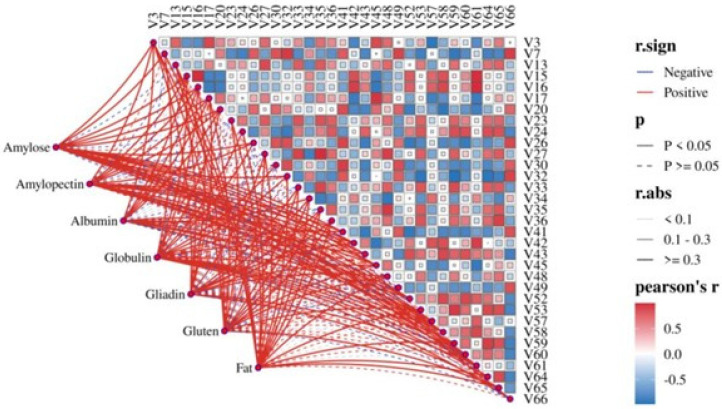
Correlation of differential volatiles in *Daqu* with major components of wheat.

To quantitatively reveal the associations between major wheat components and the aforementioned key differential volatile compounds, Mantel Test correlation analysis was performed (Figure 6). The results showed: amylose showed positive correlations with 27 differential volatiles, including 16 significant positive correlations (e.g., 2-butanol, 2,5-dimethylpyrazine, ethyl butyrate; *p* < 0.05). Amylopectin showed positive correlations with 26 volatiles, including 16 significant positive correlations (e.g., n-amyl alcohol, isovaleric acid, propyl acetate; *p* < 0.05). Among the protein components, globulin showed the strongest positive correlations with volatiles (25 positive correlations, 17 of which were significant, e.g., hexanoic acid, 3-methylpentanoic acid, benzyl alcohol; *p* < 0.05), while the non-gluten protein albumin showed fewer positive correlations (20, 12 significant). Gluten proteins (gliadin and glutenin) exhibited similar correlation patterns with volatiles. Despite its lower abundance in wheat, fat showed positive correlations with 30 differential volatiles (16 of which were significantly positive, e.g., isobutanol, 2-furaldehyde, hexanal; *p* < 0.05), indicating its significant influence on the formation of flavor compounds in *Daqu*.

Overall, differences in the chemical composition of wheat raw materials regulated microbial metabolism, altered the composition of volatile components, and ultimately resulted in significant variations. The PLS-DA model clearly confirmed the holistic nature of this variation, while the Mantel Test analysis statistically quantified the intrinsic links between specific wheat components and numerous key flavor compounds, further strengthening the above conclusion.

## 4. Conclusions

This study demonstrates that the choice of wheat variety is a decisive factor in shaping the functional characteristics and flavor potential of Daqu, primarily through its influence on the underlying microbial metabolism. Our findings not only confirm the established link between raw materials and Daqu quality [22], but also provide deeper, mechanistic insights. We found that the high starch content in CM605 wheat creates a metabolic environment conducive to ester and ketone formation, aligning with the known requirement for abundant carbon sources in these pathways [51]. However, beyond this general principle, our analysis revealed more nuanced relationships. For instance, the specific protein composition, particularly the high glutenin content in NM660 wheat, emerged as a more critical driver for the generation of alcohols and Strecker aldehydes than total protein content alone. Furthermore, we observed a previously overlooked trade-off in CM605 Daqu, where high acidity, likely a result of vigorous starch fermentation, appears to coincide with suppressed liquefying power-a consideration vital for process design.

Perhaps most notably, we identified several variety-specific volatile markers, such as 1-octen-3-one and vanillin for CM605 Daqu and ethyl laurate for NM660 Daqu. These compounds offer a direct, traceable link from raw material to product aroma, providing practical tools for quality control and targeted *Baijiu* production.

In practical terms, CM605 Daqu, with its dominant ester and ketone profile, is ideally suited for producing *Baijiu* where fruity and fragrant aromas are desired. NM660 Daqu, rich in diverse alcohols and aldehydes and possessing strong saccharification power, is optimal for processes requiring robust flavor complexity and high fermentation efficiency. Meanwhile, MM907 Daqu, with its superior liquefaction power and unique production of 1-hexanol, provides a foundation for crafting *Baijiu* with a distinct, lipid-derived character.

Finally, we acknowledge that this study, while elucidating the chemical outcomes, does not fully unravel the microbial mechanisms behind them. The core microbial communities and their succession patterns remain a black box. Therefore, future work employing high-throughput sequencing and multi-omics technologies is essential to directly link these wheat-specific compositions to the functional microbiota they nurture, ultimately completing our understanding of the “raw material-microbiota-quality” axis in Daqu fermentation.

Although this study revealed the significant effects of wheat composition on *Daqu*’s volatile and physicochemical properties, it has limitations. The primary limitation is the insufficient analysis of the microbial community. This study focused on the association between raw material properties and the final products but did not deeply analyze the structure and succession patterns of the core microbiota (bacteria, fungi, yeasts) developed during *Daqu*-making. It should be noted that this study focused on an in-depth comparison of three wheats with representative chemical profiles. While this provides powerful mechanistic insights into the key role of raw material composition, future studies incorporating a wider range of wheat samples are warranted to validate and extend these findings and to build more generalized models.

Based on these findings and shortcomings, future research should employ high-throughput sequencing (16S/ITS rRNA amplicon sequencing) combined with metagenomic and metatranscriptomic technologies. This will allow for a systematic analysis of how different wheat raw materials shape the microbial community structure throughout the *Daqu*-making process and help identify key functional microbial groups and their metabolic activities. Future studies incorporating sensory analysis and calculation of Odor Activity Values will be crucial to directly link these compositional differences to the perceived flavor profile of the final *Baijiu*.

## Figures and Tables

**Table 1:** *Daqu* Sensory Evaluation Criteria.

Parameter (Max Score)	First-Grade *Daqu*	Second-Grade *Daqu*	Third-Grade *Daqu*
Appearance (20)	Gray-white, uniform mold coverage, no cracks or fungal spots (15–20)	Gray-white, relatively uniform mold, no cracks/fungal spots (10–15)	Gray/yellowish, basic mold coverage, minor cracks/fungal spots (≤10)
Aroma (20)	Intense pure fermentation aroma (15–20)	Moderate pure aroma (10–15)	Weak aroma with off-odors (≤10)
Cross-Section (20)	Uniform texture, porous, healthy hyphae, gray/red-yellow spots, no unfermented core or water rings (15–20)	Same description as First-grade (10–15)	Irregular texture, gray/red/yellow spots, minor unfermented core/water rings permitted (≤15)
Shell thickness (20)	≤0.5 cm (15–20)	≤1.0 cm (10–15)	>1.0 cm (≤10)
Comprehensive score (20)	17–20	14–17	12–14

**Table 2:** Characterization indicators of wheat samples.

Wheat Variety	Dry Basis Thousand Kernel Weight (g)	Test Weight (g/L)	Hardness Index (%)	Wet Gluten (%)
MM907	45.96 ± 0.61 b	786.00 ± 1.00 b	46.51 ± 0.39 b	25.12 ± 0.32 b
CM605	44.44 ± 0.26 b	825.67 ± 0.58 a	44.24 ± 0.14 c	24.13 ± 0.41 c
NM660	50.59 ± 1.21 a	823.67 ± 1.53 a	48.43 ± 1.18 a	26.34 ± 0.22 a
LSD (α = 0.05)	1.59	2.22	1.44	0.66

Note: Data with different letters (a, b, c) within each column are significantly different (*p* < 0.05).

**Table 3:** Main properties of the produced *Daqu*.

Wheat Variety	Total Starch (%)	Amylose (%)	Amylopectin (%)	Albumin (mg/g)	Globulin (mg/g)	Gliadin (mg/g)	Glutenin (mg/g)	Total Protein (mg/g)	Fat (%)	Moisture (%)
MM907	64.64 ± 1.07 b	17.31 ± 0.28 c	82.69 ± 0.28 a	25.7 ± 1.43 a	16.92 ± 0.54 a	36.94 ± 1.54 b	47.61 ± 0.62 b	127.16 ± 1.07 b	1.89 ± 0.019 a	13.91 ± 0.22 a
CM605	67.97 ± 1.16 a	26.04 ± 0.57 a	73.76 ± 0.57 c	18.68 ± 0.65 b	14.82 ± 0.28 c	34.41 ± 1.69 b	45.38 ± 1.34 b	113.30 ± 2.74 c	1.44 ± 0.06 b	12.10 ± 0.00 c
NM660	63.21 ± 0.98 b	20.07 ± 0.54 b	79.93 ± 0.54 b	27.84 ± 0.92 a	15.73 ± 0.19 b	41.84 ± 0.22 a	54.74 ± 1.57 a	140.15 ± 2.51 a	0.64 ± 0.025 c	12.42 ± 0.02 b
LSD (α = 0.05)	2.15	0.96	0.96	2.73	0.74	2.65	2.48	4.46	0.08	0.03

Note: Data with different letters (a, b, c) within each column are significantly different (*p* < 0.05).

**Table 4:** Physicochemical indexes of *Daqu* made from different wheat varieties.

*Daqu* Sample	Moisture (%)	Acidity (mmol/10 g)	Amino Acid Nitrogen (g/kg)	Saccharification Power (mg/g·h)	Liquefaction Power (g/g·h)
MM907	14.76 ± 0.08 a	0.70 ± 0.00 b	3.28 ± 0.05 c	582 ± 8.49 b	1.90 ± 0.03 a
CM605	13.94 ± 0.01 b	0.93 ± 0.00 a	3.52 ± 0.01 b	549 ± 4.24 b	1.12 ± 0.01 c
NM660	12.21 ± 0.18 c	0.63 ± 0.00 c	4.59 ± 0.06 a	756 ± 16.97 a	1.67 ± 0.02 b
LSD (α = 0.05)	0.23	0.00	0.09	22.45	0.04

Note: Data with different letters (a, b, c) within each column are significantly different (*p* < 0.05).

**Table 5:** Volatile compounds in different *Daqu* samples from different wheat varieties (μg/g).

No.	Volatile Compounds	CM605	MM907	NM660
Esters				
V1	Ethyl acetate	3.23 ± 0.41 a	4.12 ± 0.22 b	3.89 ± 0.15 b
V2	Ethyl hexanoate	33.45 ± 1.23 a	18.81 ± 2.78 b	25.34 ± 1.36 c
V3	Ethyl butyrate	14.75 ± 1.12 a	17.63 ± 0.89 b	20.22 ± 1.22 c
V4	Ethyl linoleate	0.46 ± 0.17 a	0.54 ± 0.08 b	0.43 ± 0.11 c
V5	Ethyl oleate	0.25 ± 0.04 a	0.35 ± 0.08 b	0.31 ± 0.06 ab
V6	Ethyl propionate	17.14 ± 1.01 a	15.53 ± 0.45 b	14.68 ± 0.44 b
V7	Ethyl palmitate	0.86 ± 0.09 a	1.54 ± 0.22 b	0.61 ± 0.08 c
V8	Ethyl octanoate	6.72 ± 1.56 a	3.24 ± 0.23 b	5.78 ± 0.14 a
V9	Ethyl 2-methylbutanoate	4.25 ± 1.02 a	7.47 ± 0.25 b	6.74 ± 1.87 b
V10	Phenethyl acetate	2.47 ± 0.30 a	3.16 ± 0.44 b	2.68 ± 0.21 a
V11	Pentyl propanoate	0.36 ± 0.05 a	0.14 ± 0.11 b	0.08 ± 0.02 b
V12	Ethyl phenylacetate	6.78 ± 2.21 a	7.24 ± 0.36 ab	5.41 ± 0.74 b
V13	Ethyl laurate	-	-	0.32 ± 0.21
V14	Ethyl benzoate	2.31 ± 0.33 a	1.14 ± 0.07 b	1.52 ± 0.36 b
V15	Isoamyl acetate	0.22 ± 0.06	-	-
V16	Propyl acetate	1.95 ± 0.26	-	-
Alcohols				
V17	n-Pentanol	8.03 ± 1.24 a	15.31 ± 2.13 b	17.86 ± 1.02 c
V18	2-Phenylethanol	1.35 ± 0.21 a	1.67 ± 0.08 a	1.41 ± 0.33 a
V19	2,3-Butanediol	0.15 ± 0.03 a	0.08 ± 0.02 b	0.07 ± 0.04 b
V20	2-Butanol	-	0.58 ± 0.11 a	0.35 ± 0.07 b
V21	1-Octen-3-ol	6.23 ± 1.44 a	2.53 ± 0.24 b	4.34 ± 0.35 c
V22	3-Methylbenzyl alcohol	0.04 ± 0.02 a	0.11 ± 0.04 b	0.08 ± 0.03 ab
V23	Isobutanol	0.08 ± 0.01 a	0.06 ± 0.02 a	0.12 ± 0.03 b
V24	Heptanol	0.31 ± 0.10 a	-	0.47 ± 0.13 b
V25	n-Hexanol	0.48 ± 0.09 a	0.32 ± 0.10 b	0.27 ± 0.08 b
V26	Benzyl alcohol	2.33 ± 0.11 a	3.27 ± 0.21 b	2.46 ± 0.14 a
V27	Furfuryl alcohol	-	-	0.11 ± 0.04
V28	3-Furanmethanol	0.09 ± 0.03	-	-
V29	Linalool	0.37 ± 0.06 a	0.21 ± 0.04 b	0.18 ± 0.03 b
V30	1-Hexanol	-	0.16 ± 0.07	-
V31	n-Heptanol	0.08 ± 0.02 a	0.13 ± 0.04 ab	0.17 ± 0.05 b
Aldehydes				
V32	Benzaldehyde	12.87 ± 0.09 a	15.86 ± 0.14 b	11.43 ± 0.16 c
V33	2-Furaldehyde	0.08 ± 0.01 a	0.05 ± 0.03 a	0.15 ± 0.04 b
V34	Furfural	0.18 ± 0.03 a	0.16 ± 0.05 a	-
V35	3-Methyl-2-butenal	-	-	6.58 ± 2.14
V36	Hexanal	4.62 ± 1.03 a	2.88 ± 0.25 b	9.43 ± 2.44 c
V37	Decanal	0.32 ± 0.15	-	-
V38	Nonanal	0.17 ± 0.05 a	0.43 ± 0.11 b	0.25 ± 0.08 a
V39	Pyrrole-2-carbaldehyde	0.06 ± 0.02	-	-
Acids				
V40	Acetic acid	0.63 ± 0.13 a	0.54 ± 0.04 ab	0.47 ± 0.06 b
V41	Hexanoic acid	1.55 ± 0.08 a	2.63 ± 0.17 b	1.79 ± 0.20 a
V42	Isovaleric acid	1.86 ± 0.32 a	0.77 ± 0.12 b	0.64 ± 0.04 b
V43	3-Methylpentanoic acid	0.59 ± 0.14 a	-	0.44 ± 0.08 b
V44	Propanoic acid	0.41 ± 0.21	-	-
V45	Butanoic acid	0.49 ± 0.14 a	1.32 ± 0.22 b	1.55 ± 0.07 c
Phenolics				
V46	2,4-Di-tert-butylphenol	0.12 ± 0.04 a	0.11 ± 0.05 a	0.18 ± 0.04 b
V47	4-Ethylguaiacol	0.03 ± 0.02 a	0.04 ± 0.01 a	0.03 ± 0.02 a
V48	4-Vinylguaiacol	0.09 ± 0.05 a	0.14 ± 0.02 a	0.31 ± 0.06 b
V49	p-Vinylguaiacol	-	0.05 ± 0.03	-
V50	Vanillin	0.03 ± 0.02	-	-
V51	Vinylguaiacol	-	0.02 ± 0.01 a	0.02 ± 0.01 a
Ketones				
V52	2-Octanone	4.87 ± 0.35 a	3.18 ± 0.18 b	4.15 ± 0.22 c
V53	2-Heptanone	1.68 ± 0.11 a	1.14 ± 0.20 b	2.12 ± 0.14 c
V54	1-Octen-3-one	0.36 ± 0.13	-	-
V55	Acetophenone	0.23 ± 0.07 a	0.34 ± 0.12 a	0.26 ± 0.05 a
Pyrazines				
V56	2-Methylpyrazine	0.33 ± 0.13 a	0.24 ± 0.04 a	0.28 ± 0.11 a
V57	2,3-Dimethylpyrazine	0.74 ± 0.13 a	0.51 ± 0.06 b	0.38 ± 0.07 c
V58	2,5-Dimethylpyrazine	2.14 ± 0.13 a	1.12 ± 0.09 b	1.53 ± 0.03 c
V59	2,6-Dimethylpyrazine	1.43 ± 0.07 a	0.89 ± 0.05 b	1.76 ± 0.16 c
V60	2,3,5-Trimethylpyrazine	0.08 ± 0.02 a	-	0.07 ± 0.03 a
V61	2,3,5,6-Tetramethylpyrazine	1.07 ± 0.12	-	-
V62	2-Ethyl-5-methylpyrazine	-	0.03 ± 0.02	-
V63	3-Ethyl-2,5-dimethylpyrazine	0.02 ± 0.01 a	-	0.04 ± 0.02 a
Others				
V64	Tetramethylpyrazine	0.22 ± 0.08 a	-	0.34 ± 0.05 b
V65	2-(n-Pentyl)furan	1.18 ± 0.12 a	0.78 ± 0.11 b	2.04 ± 0.21 c
V66	2-Acetylpyrrole	-	0.24 ± 0.07	-

Note: “-” means not detected. Data with different letters (a, b, c) within each row are significantly different (*p* < 0.05).

## Data Availability

The original contributions presented in the study are included in the article, further inquiries can be directed to the corresponding authors.

## Supplementary Materials

The following supporting information can be downloaded at: https://www.mdpi.com/article/10.3390/foods14213638/s1, Table S1: Sensory evaluation scores of Daqu produced from different wheat varieties.
