# Supplementary material for: Effects of Wheat Composition on the Physicochemical and Volatile Components of Daqu (A Primary Starter for Chinese Baijiu Fermentation)
Source: Foods. 2025 Oct 24;14(21):3638. doi: 10.3390/foods14213638 (PMC12610647; doi:10.3390/foods14213638)
Supplement: Supplementary file 1 [file foods-14-03638-s001.zip › foods-3900476-supplementary.pdf]

Table S1. Sensory evaluation scores of *Daqu* produced from different wheat varieties

Daqu Sample	Appearance	Aroma	Cross-Section	Shell Thickness	Comprehensive Score
MM907	16.00 ± 0.71b	17.00 ± 0.00a	17.80 ± 0.45a	14.40 ± 0.55a	16.80 ± 0.84a
CM605	15.40 ± 0.55b	16.20 ± 0.48b	16.80 ± 0.45b	14.60 ± 1.34a	16.00 ± 0.71b
NM660	16.80 ± 0.48a	16.60 ± 0.55b	17.40 ± 0.89ab	15.00 ± 1.58a	16.80 ± 0.45a
LSD(α=0.05)	0.89	0.63	0.98	1.52	0.94

Note: Data with different letters (a, b, c,) within each column are significantly different ($p < 0.05$).
